# Esophageal Tuberculosis Mimicking Malignancy in an Immunocompetent Patient: A Case Report

**DOI:** 10.7759/cureus.86606

**Published:** 2025-06-23

**Authors:** Soraya Gioftsiou, Malak Faiz, Zineb Malki, Mohamed Mohammadi

**Affiliations:** 1 Hepato-gastroenterology, Cheikh Zaid University Hospital, Rabat, MAR

**Keywords:** extrapulmonary, infection, malignancy, oesophagitis, tuberculous

## Abstract

Esophageal tuberculosis is a rare manifestation of extrapulmonary tuberculosis, often misdiagnosed due to its nonspecific clinical features and resemblance to malignancy. We report the case of a 65-year-old immunocompetent woman with no prior medical history who presented with an 8-kg weight loss over three months and progressive dysphagia. Laboratory investigations revealed leukocytosis without other abnormalities, and HIV serology was negative. Upper gastrointestinal endoscopy showed an ulcerated and exophytic lesion in the distal third of the esophagus. Histopathological examination revealed granulomatous inflammation with caseating necrosis, and PCR confirmed *Mycobacterium tuberculosis*. The patient was treated with standard anti-tuberculosis therapy, an initial two-month quadruple drug regimen followed by a four-month continuation phase with dual therapy. Marked clinical improvement was observed after one month of treatment. This case underscores the importance of considering tuberculosis in the differential diagnosis of esophageal lesions, even in immunocompetent patients, and highlights the critical role of systematic biopsy of any lesion during endoscopy, including those with typical appearances such as ulcerations.

## Introduction

Tuberculosis (TB) remains a major global health issue, especially in developing countries. According to the World Health Organization, TB is among the top 10 causes of death worldwide, with over 10 million people affected annually [1]. While pulmonary TB is the most common form, extrapulmonary manifestations account for a significant proportion of cases, particularly in immunocompromised individuals [2,3]. Among these, esophageal tuberculosis is exceedingly rare, comprising less than 0.2% of gastrointestinal TB cases [4]. It is often misdiagnosed due to its nonspecific clinical presentation and frequent mimicry of esophageal carcinoma [5,6]. Here, we report a case of primary esophageal TB in a 65-year-old immunocompetent woman presenting with weight loss and progressive dysphagia.

## Case presentation

A 65-year-old woman with no significant past medical history and from a low socioeconomic background presented with an 8-kg weight loss over three months, corresponding to approximately 13% of her body weight, along with intermittent and progressive dysphagia to solids accompanied by fatigue. She denied fever, cough, night sweats, or other systemic symptoms. Physical examination was unremarkable, and her vital signs were stable.

Initial laboratory workup revealed a white blood cell count of 12,800/mm³ and an elevated C-reactive protein (CRP) level of 36 mg/L, suggesting an inflammatory process. All other parameters, including liver and kidney function, electrolytes, and hemoglobin, were within normal limits. HIV serology was negative.

**Figure 1:**
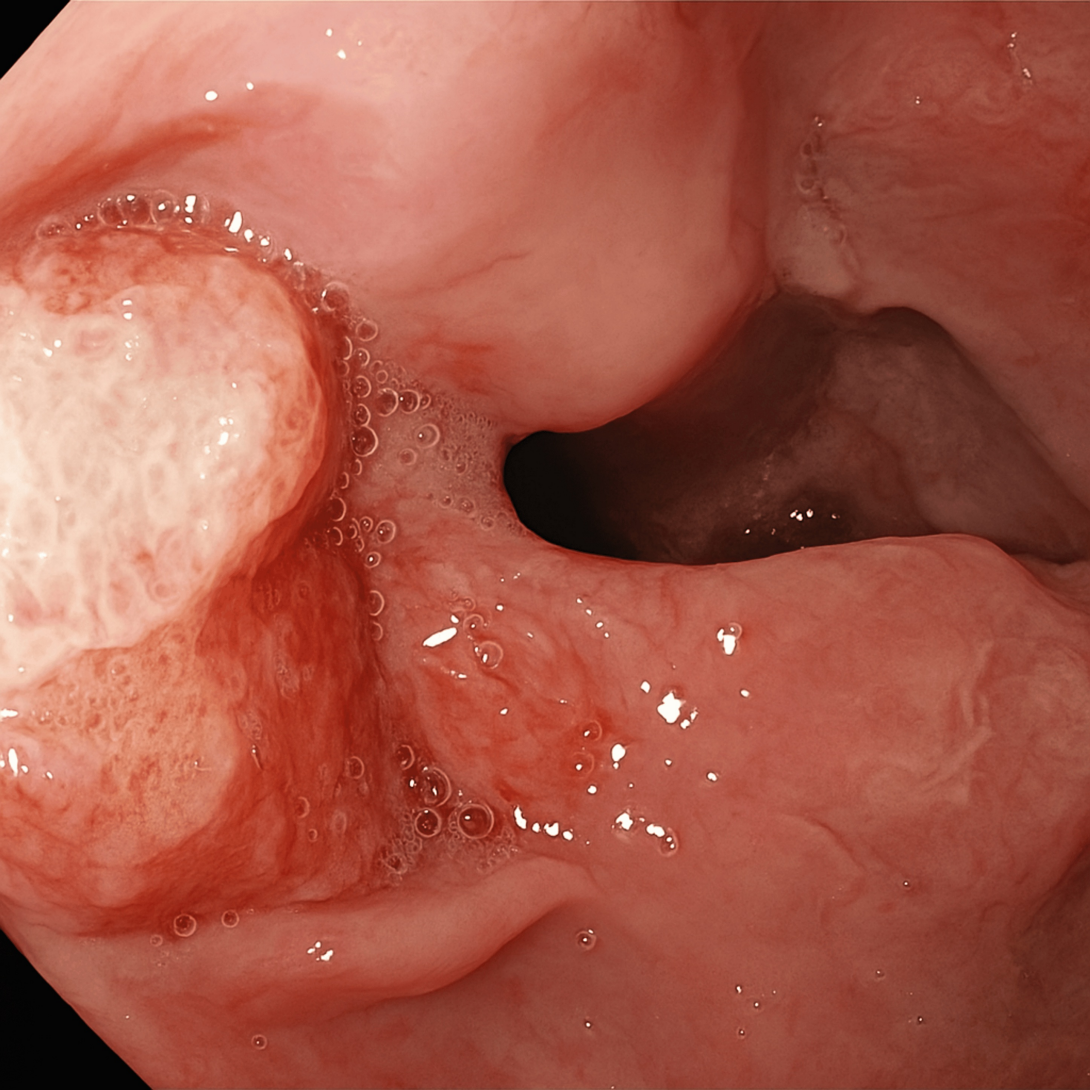
Endoscopic view of an ulcerated and exophytic lesion in the lower third of the esophagus Endoscopic image showing a large, ulcerated, exophytic lesion with an irregular and friable surface located in the distal third of the esophagus.

An upper gastrointestinal endoscopy was performed due to progressive dysphagia and clinical concern for malignancy. It revealed an ulcerated, exophytic lesion in the lower third of the esophagus at 28 cm from the incisors (Figure 1). Multiple biopsies were taken from the lesion and from unaffected areas of the esophagus, two from the lower third and two from the upper third, to help exclude differential diagnoses.

Acid-fast bacilli (AFB) staining initially confirmed the presence of mycobacteria. However, as this test does not distinguish *Mycobacterium tuberculosis* from other nontuberculous mycobacteria, PCR testing was performed and confirmed the presence of *Mycobacterium tuberculosis*.

**Figure 2:**
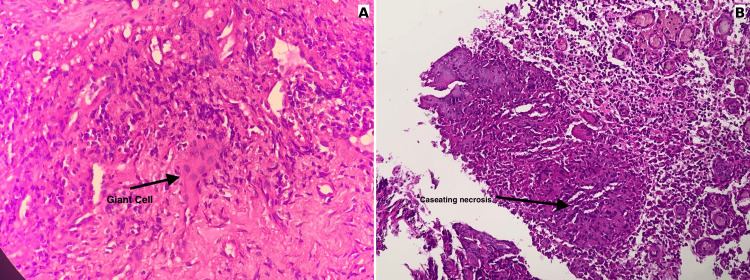
Histopathological features of esophageal tuberculosis A. Hematoxylin and eosin (H&E) stained section showing a Langhans-type giant cell within granulomatous inflammation (400x). B. H&E stained section demonstrating caseating necrosis, a hallmark of tuberculosis (400x).

Histological analysis revealed granulomatous inflammation with epithelioid giant cells and central caseating necrosis, features that are pathognomonic for tuberculosis (Figure 2). Although this was sufficient to confirm the diagnosis, the interferon-gamma release assay (IGRA) was also positive (0.54 IU/mL), further supporting the diagnosis.

Differential diagnoses such as Crohn’s disease and sarcoidosis, which can also present with granulomatous inflammation and epithelioid giant cells but typically without caseating necrosis, were ruled out based on histological findings.

Given the rarity of esophageal tuberculosis, further investigations were conducted to exclude other sites of *Mycobacterium tuberculosis* infection, particularly in the respiratory tract. A chest X-ray was normal, and three consecutive sputum samples were negative for tuberculosis. 

We concluded that this was a case of primary esophageal tuberculosis, most likely caused by the ingestion of contaminated food.

The patient was started on standard anti-tuberculosis therapy, consisting of isoniazid, rifampicin, pyrazinamide, and ethambutol for an initial intensive phase of two months, followed by a continuation phase with isoniazid and rifampicin for an additional four months.

No one in the patient’s household exhibited symptoms of pulmonary or extrapulmonary TB, and no specific risk factors were identified aside from her low socioeconomic status and residence in an endemic region.

After just one month of treatment, the patient reported significant improvement in dysphagia. By the two-month follow-up, she had gained 4 kg, and dysphagia had completely resolved. A follow-up endoscopy performed after completion of therapy was strictly normal, with no residual signs of the initial lesion.

## Discussion

Gastrointestinal tuberculosis is a relatively uncommon form of extrapulmonary TB, typically considered within the broader category of abdominal tuberculosis, which ranks among the top three to four most frequent sites of extrapulmonary involvement after lymphatic, pleural, and skeletal forms. Esophageal tuberculosis, in particular, is exceedingly rare and usually results from contiguous spread from mediastinal lymph nodes, direct extension from adjacent structures, or, more rarely, primary infection via ingestion. Clinical presentation often includes dysphagia, odynophagia, retrosternal pain, and weight loss, all of which may mimic esophageal carcinoma [1,2].

Diagnosis is often challenging due to the rarity of the condition and the nonspecific endoscopic findings. Histopathological confirmation with identification of caseating granulomas and positive AFB staining remains the cornerstone of diagnosis. Molecular techniques such as PCR have improved diagnostic sensitivity, especially in paucibacillary lesions [3,4].

This case also highlights an essential procedural point: even when endoscopic findings appear to be "typical" ulcerations or nodular lesions, systematic biopsy is crucial. Without histological sampling, rare entities like tuberculous esophagitis may be misdiagnosed or missed entirely. The decision to biopsy should remain standard practice, particularly when lesions raise even minimal concern for malignancy or infection [3,5,6].

The standard treatment follows the same regimen as pulmonary TB. Most patients respond well, with resolution of symptoms and healing of esophageal lesions. Surgery is rarely needed and is reserved for complications such as strictures or fistulas [2,5,7].

This case emphasizes the importance of considering TB in the differential diagnosis of esophageal lesions, even in the absence of pulmonary involvement or known immunosuppression. Early recognition and treatment can lead to complete clinical recovery and prevent unnecessary surgical intervention or misdiagnosis as cancer [6,7].

## Conclusions

This case highlights a rare presentation of primary esophageal tuberculosis in an elderly immunocompetent woman, initially mimicking esophageal cancer. Histopathological and molecular confirmation allowed prompt initiation of standard anti-TB therapy, resulting in rapid symptom resolution, 4 kg weight gain within two months, and complete endoscopic healing. The absence of pulmonary involvement supports a likely oral route of transmission through contaminated food. This case underscores the importance of considering TB in atypical esophageal lesions, particularly in endemic areas, and reinforces the value of routine biopsy during endoscopy to avoid misdiagnosis.
